# Lipschütz Ulcers: Classic Presentation of an Uncommon Condition

**DOI:** 10.7759/cureus.38505

**Published:** 2023-05-03

**Authors:** Carter Gay, Colby Kihara, Austin Haley, Arsh N Patel, Laurence Stolzenberg, Erika Haviland, Steve Shassberger

**Affiliations:** 1 Research, Alabama College of Osteopathic Medicine, Dothan, USA; 2 Obstetrics and Gynecology, Athens-Limestone Hospital, Athens, USA

**Keywords:** lipschütz ulcers, vulvar lesions, mycoplasma pneumoniae, epstein barr virus, genital ulcers

## Abstract

We describe the case of a 17-year-old female who presented with the acute onset of painful genital ulcers after experiencing a week of flu-like symptoms. A physical exam revealed two 1 cm necrotic ulcers on the right vulva with an erythematous margin and overlying exudate. A diagnosis of Lipschütz ulcers was made based on the classic signs and symptoms, in addition to ruling out relevant infectious and inflammatory diseases. Our goal in presenting this case is to add to the literature and increase awareness regarding this uncommon condition. The differential diagnosis and workup for genital ulcers can be extensive, but when diagnosed correctly, treatment and reassurance can provide great comfort for the patient.

## Introduction

Lipschütz ulcers are an uncommon presentation of painful genital ulcers, most often in adolescent females, that were first described in 1913 by dermatologist Benjamin Lipschütz [1]. The onset is acute and classically follows a preceding viral illness, most commonly Epstein-Barr virus (EBV), but can also occur after a bacterial infection, like *Mycoplasma pneumoniae* [2]. Lesions are generally confined to the vulva and can be unilateral or bilateral. The gross appearance is described as a necrotic center with an overlying gray-black exudate and an erythematous, well-demarcated border. The disease course is typically self-limited over the course of two to three weeks, but pain management, including topical anesthetics or oral analgesics, is often appropriate [3]. Here, we present a 17-year-old female who developed unilateral ulcerations on the vulva a week following an upper respiratory illness.

## Case presentation

A 17-year-old female presented to the clinic with a chief complaint of genital lesions. Over the past week, the patient had experienced symptoms of fever, chest congestion, cough, and intermittent shortness of breath, in addition to dysuria. These symptoms prompted the patient to present herself to the ER, where she was diagnosed with pneumonia and a urinary tract infection. At that time, she was started on cefdinir 300 mg twice daily, doxycycline 100 mg twice daily, and oral prednisone 20 mg daily.

**Figure 1:**
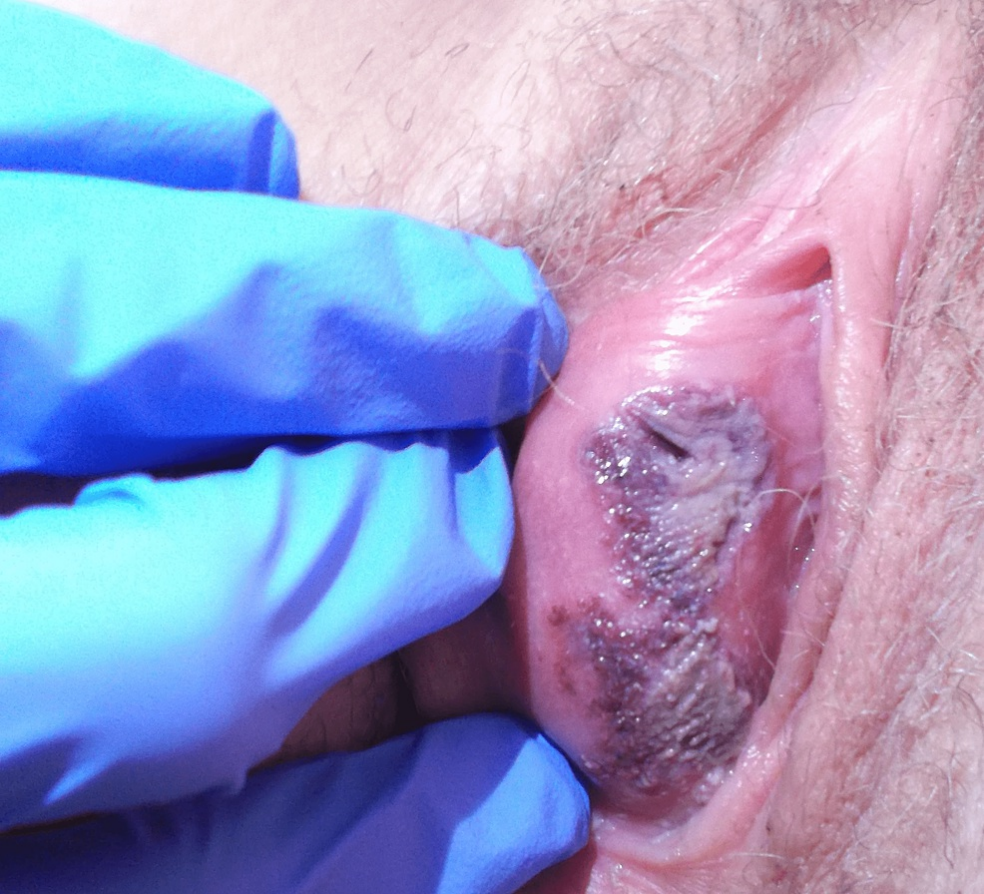
Two necrotic ulcers on the right vulva with overlying exudate. The lesions are surrounded by an erythematous border and have notable swelling underneath.

After the development of her respiratory symptoms, the patient noted the acute onset of intensely painful genital lesions on the right vulva (Figure 1). Upon inspection, the lesions were soft and exquisitely tender to palpation. No inguinal lymphadenopathy was present. A speculum exam revealed a normal-appearing cervix with minimal discharge that was negative on wet prep. There was no history of sexual abuse or physical trauma. The patient reported oral and vaginal sex with consistent condom use and denied any known exposure to sexually transmitted diseases (STDs). She also denied tampon use and reported that pads are changed regularly when menstruating. Periods were regular with moderate flow, occurring every 28 days and lasting five to seven days. The combined workup from the outpatient clinic and emergency department was extensive, but all results were negative. Testing included influenza, severe acute respiratory syndrome coronavirus-2 (SARS-CoV-2), human immunodeficiency virus-1/2 (HIV-1/2), Epstein-Barr virus (EBV), cytomegalovirus (CMV), herpes simplex virus-1/2 (HSV-1/2), hepatitis A, hepatitis B, hepatitis C, and syphilis.

Based on the clinical presentation, the physical appearance of the ulcers, and the exclusion of other relevant etiologies, a diagnosis of Lipschütz ulcers was recommended. Due to the significant pain associated with the ulcers, the patient required 2% topical lidocaine for symptomatic relief. A follow-up at six weeks revealed the ulcers had resolved, but there remained some residual skin sensitivity and inflammation.

## Discussion

The differential diagnosis regarding genital ulcers is extensive, even in adolescents. Common culprits include sexually transmitted infections, inflammatory reactions, and autoimmune diseases. Lipschütz ulcers are a lesser-known entity that most often presents in females as single or multiple painful genital ulcers. Lipschütz ulcers may occur in women of any age, but based on a review of 158 known cases, approximately 90% were ≤ 20 years of age [2].

The clinical presentation classically involves a prodrome of flu-like symptoms before the acute onset of genital ulcerations. While the underlying etiology is poorly understood, there is strong evidence to suggest a link to viral illnesses, mainly Epstein-Barr virus (EBV) [4]. Many other viruses have also been linked, including influenza, cytomegalovirus (CMV), and paramyxovirus. Cases involving Lipschütz ulcers preceded by bacterial infections have also been documented and often include *Mycoplasma pneumoniae*, group A *Streptococcus*, *Salmonella,* and *Toxoplasma* [5]. One proposed hypothesis regarding the pathogenesis involves a hypersensitivity reaction with subsequent deposition of immune complexes and resultant small vessel thrombosis affecting the vulvar region [1].

As for the gross appearance of the vulvar lesions, common findings include necrotic ulcers with an erythematous border and an overlying gray-black exudate. The ulcers are usually at least 1 cm in width and relatively deep [3].

The diagnosis is often made based on clinical judgment and the exclusion of other more common etiologies of genital ulcers. In terms of diagnostic criteria, a literature review of 60 documented cases was conducted, with subsequent proposals of major and minor criteria to guide the clinical diagnosis of Lipschütz ulcers. Major criteria include: 1) acute onset of one or more painful ulcerous lesions in the vulvar region; 2) exclusion of other potential causes of the ulcer, including infectious and non-infectious processes. Minor criteria include: 1) ulcers confined to the vestibule or labia minora; 2) no history of sexual intercourse or no sexual intercourse within the last 3 months; 3) flu-like symptoms; 4) systemic infection within the last two to four weeks prior to the onset of the genital ulcer. Diagnosis requires both major criteria and at least two minor criteria to recommend the diagnosis of Lipschütz ulcer [6].

Due to a lack of awareness, misdiagnosis can lead to unnecessary diagnostic testing and treatment. Furthermore, the extensive workup may cause undue distress for the patient if a label is placed regarding sexually transmitted diseases, inflammatory diseases, or sexual abuse. An effort should be made to emphasize the fact that no evidence exists to suggest a relationship between Lipschütz ulcers and sexually transmitted infections [7].

Additionally, neoplastic processes are an important component of the differential diagnosis of any patient presenting with genital ulcerations. However, due to the occurrence of Lipschütz ulcers in younger age groups and the relatively short duration before self-resolution, biopsies are not typically required. Furthermore, histopathological examination in several reported cases only demonstrated a non-specific inflammatory cell infiltrate. In some instances, based on clinical judgment, with lesions persisting past four weeks or with significant risk factors for neoplasia, a biopsy may be appropriate [8].

Once the diagnosis has been made, treatment typically involves topical anesthetics or oral analgesics with spontaneous resolution of the ulcers over the course of two to four weeks. For more severe cases, systemic corticosteroids may be appropriate [9]. Although self-limiting, there have been documented cases of recurrence, but frequent episodes may warrant evaluation for complex aphthosis or other ulcerative syndromes [10,11].

## Conclusions

Lipschütz ulcers are a rare but clinically significant cause of genital ulcers in adolescent females. In this case, we present a 17-year-old female who developed unilateral ulcerations on the vulva following an upper respiratory illness. Lipschütz ulcers should be included in the differential diagnosis for genital ulcers. Furthermore, increased awareness of this entity is needed to allow for proper management and to avoid unnecessary workups and incorrect treatment that may be costly and distressing for the patient.
